# Fabrication of highly effective hybrid biofuel cell based on integral colloidal platinum and bilirubin oxidase on gold support

**DOI:** 10.1038/s41598-018-34740-w

**Published:** 2018-11-05

**Authors:** Md Qumrul Hasan, Robinson Kuis, J. Shankara Narayanan, Gymama Slaughter

**Affiliations:** University of Maryland Baltimore County and Bioelectronics Laboratory, Department of Computer Science and Electrical Engineering, Maryland, USA

## Abstract

A hybrid biofuel cell (HBFC) is explored as a low-cost alternative to abiotic and enzymatic biofuel cells. Here the HBFC provides an enzymeless approach for the fabrication of the anodic electrode while employing an enzymatic approach for the fabrication of the cathodic electrode to develop energy harvesting platform to power bioelectronic devices. The anode employed 250 μm braided gold wire modified with colloidal platinum (Au-co-Pt) and bilirubin oxidase (BODx) modified gold coated Buckypaper (BP-Au-BODx) cathode. The functionalization of the gold coated multi-walled carbon nanotube (MWCNT) structures of the BP electrodes is achieved by 3-mercaptopropionic acid surface modification to possess negatively charged carboxylic groups and subsequently followed by EDC/Sulfo-NHS (1-Ethyl-3-(3-dimethylaminopropyl) carbodiimide hydrochloride and N-Hydroxysulfosuccinimide) crosslinking with BODx. The integration of the BODx and gold coated MWCNTs is evaluated for bioelectrocatalytic activity. The Au-co-Pt and BP-Au-BODx exhibited excellent electrocatalytic activity towards glucose oxidation with a linear dynamic range up to 20 mM glucose and molecular oxygen reduction, respectively. The HBFC demonstrated excellent performance with the largest open circuit voltages of 0.735 V and power density of 46.31 μW/cm^2^ in 3 mM glucose. In addition, the HBFC operating on 3 mM glucose exhibited excellent uninterrupted operational stability while continuously powering a small electronic device. These results provide great opportunities for implementing this simple but efficient HBFC to harvest the biochemical energy of target fuel(s) in diverse medical and environmental applications.

## Introduction

Implantable technologies have garnered great attention due to their ability to prolong the life of an individual suffering from a disease and/or complications associated with a disease^1^. In addition, implantable devices are capable of enabling the continuous monitoring of critical blood metabolites such as glucose to help maintain tight control of blood glucose in individual suffering from the disease diabetes. Diabetes is a debilitating disease that affects more than 33% of the people living in the United States^2^. The overarching goal of miniaturizing implantable bioelectronics such as the continuous glucose monitoring (CGM) devices still faces several challenges. Currently, electrochemical power sources such as batteries are used to power CGMs, thereby making the entire device bulky and rigid to implant. In this case, only the needle sensor is subcutaneously implanted. These batteries convert the chemical energy of a chemical reaction between two solid metallic active masses into electrical energy by consuming one of the active masses. Once one of the active masses is fully consumed, the current-producing reaction ceases, thus no current flow through the system. Moreover, batteries have limited supply of fuel and has to be recharged or replaced once all the chemical energy stored in the active mass is consumed. As technology continues to advance at a rapid pace, it is a challenge to break away from the heavy dependency on the battery as an external power source. Additionally, bioelectronic devices continue to get smaller and smaller whereas the battery technology remains relatively the same, bulky, rigid and toxic, thereby posing a challenge for their integration with implantable and wearable electronics.

Several approaches have been reported to address these challenges such as the development of low-energy electronics^3^, low-power consuming electronics^4^ and high energy density power sources^5^. A potential solution to the miniaturization limitation of current batteries for implantable devices is using biological and/or hybrid fuel cell technologies to harness the biochemical energy readily found in the body. The idea of harnessing energy directly from organic substrates by utilizing its internal chemical mechanisms to generate electrical power is considerably more stable and reliable than physical mechanisms such as mechanical motion. Biofuel cells (BFCs) are a promising ‘green’ and ‘biosafe’ alternative technology for powering implantable devices. BFCs convert chemical energy in biomolecules into electrical energy using oxidation-reduction (redox) enzymes. The most commonly used BFC comprises electrodes constructed from highly conductive multiwall carbon nanotubes (MWCNTs) that are functionalized by a hetero-bifunctional pyrenebutanoic succinimidyl ester molecule accomplished via non-covalent π-π interactions of the pyrene derivative with the pyrene on MWCNT-based electrode to immobilize glucose and oxygen selective enzymes^6,7^. Bilirubin oxidase (BODx) and laccase are the most commonly used oxygen selective enzymes^6^, wherein BODx has gained significant attention because of its ability to operate under physiological pH^8^. Pyrroloquinolinequinone-dependent glucose dehydrogenase (PQQ-GDH) and glucose oxidase or lactate dehydrogenase (LDH) are commonly used to oxidize glucose^8–12^ or lactic acid^13,14^, respectively. These enzymes are immobilized on electrodes and assembled as biofuel cell to produce electrical energy to potentially power small electronic devices. Thus, efficient generation of power from BFCs depends on the choice of anodic and cathodic material employed and the long term stability of the biocatalyst to catalyze the redox reaction.

The aforementioned factors remain key challenges to overcome when employing BFCs as a potential implantable power source. In this present study, we report the innovative hybrid biofuel cell (HBFC) that employs an enzymeless anodic electrode fabricated from 250 μm braided gold wire modified with colloidal platinum (Au-co-Pt) and covalent immobilization of BODx on gold coated Buckypaper (BP-Au-BODx) cathodic electrode. The covalent immobilization and enzyme stabilization approaches employed eliminate the non-covalent π-π stacking of pyrene on MWCNTs sidewalls and prevent the loss of enzyme activity overtime at the biocathode, thereby leading to effective enzyme immobilization on the nanotube structures. The electrochemical properties of the Au-co-Pt for glucose oxidation through the electrochemical activation of colloidal platinum and BODx for molecular oxygen reduction are examined. The resulting electrodes are used for the glucose/O_2_ HBFC construction and the performances in terms of power density and stability are evaluated. Based on our knowledge, this is the first report on the fabrication and successfully uninterrupted operation of a HBFC by preparing an enzymeless Au-co-Pt anode and covalently immobilize BODx on gold-coated carbon nanotube cathode to form an inorganic-enzyme hybrid cell. The reported HBFC can lead to the development of a low-cost alternative to abiotic and enzymatic biofuel cells that is highly effective at generating stable electrical power for bioelectronics devices. The HBFC exhibits longer operational lifetime and higher power output at low glucose concentrations (i.e., 3 mM). The results from this HBFC show the potential of such a system for energy harvesting from the body.

## Results and Discussion

### Preparation and characterization of Au-co-Pt anode

**Figure 1:**
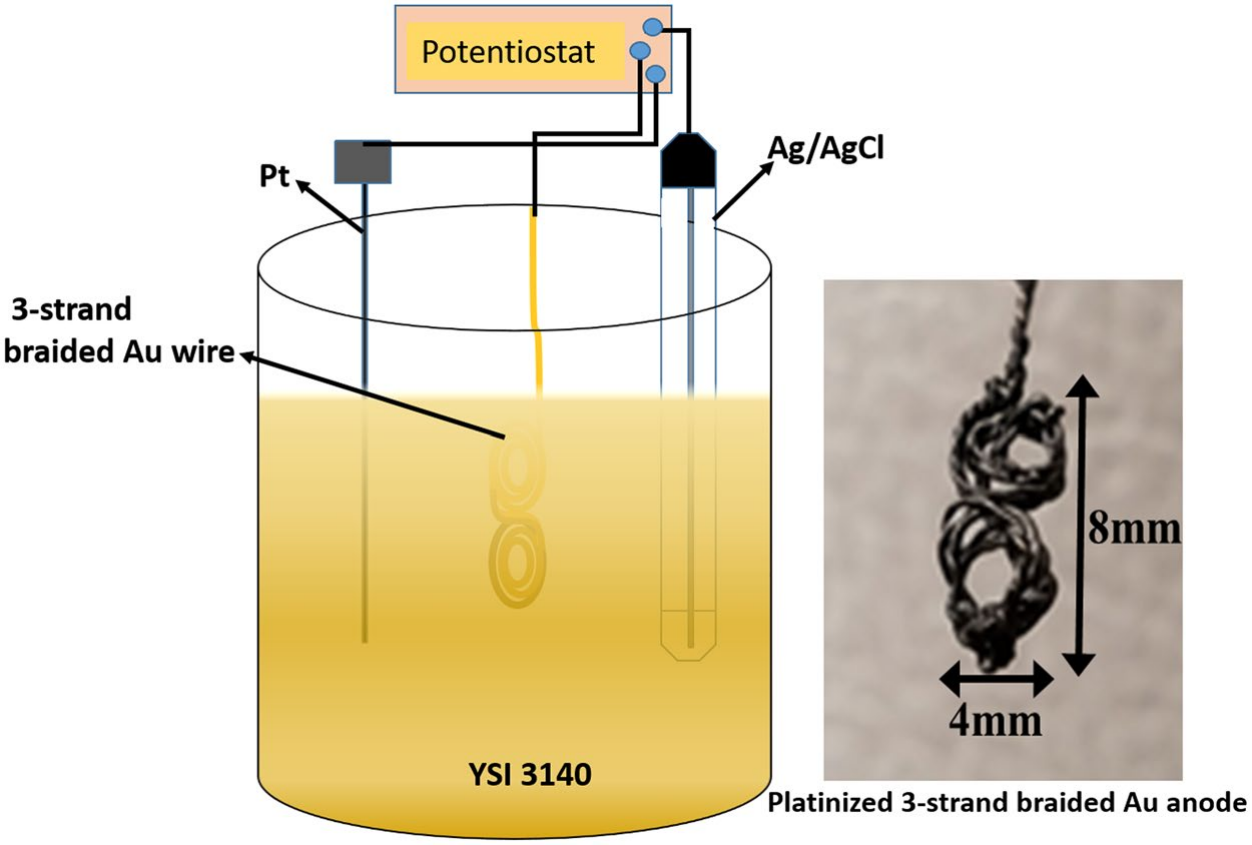
Schematic diagram of the platinization of 3-strand braided anode and optical image of the as-platinized 3-strand braided Au anode.

Figure 1 provides the overall scheme for the fabrication of the gold-colloidal-platinum (Au-co-Pt) abiotic anode and its application in the development of hybrid biofuel cell. The colloidal Pt layer was achieved by immersing 3-strand braided 250 μm Au wire in platinizing solution for the electrodeposition of platinum on Au. Instantaneous deposition has been found to occur at a potential of −225 mV, which resulted in a gradual increase in the Pt surface area that eventually levels off with time.

**Figure 2:**
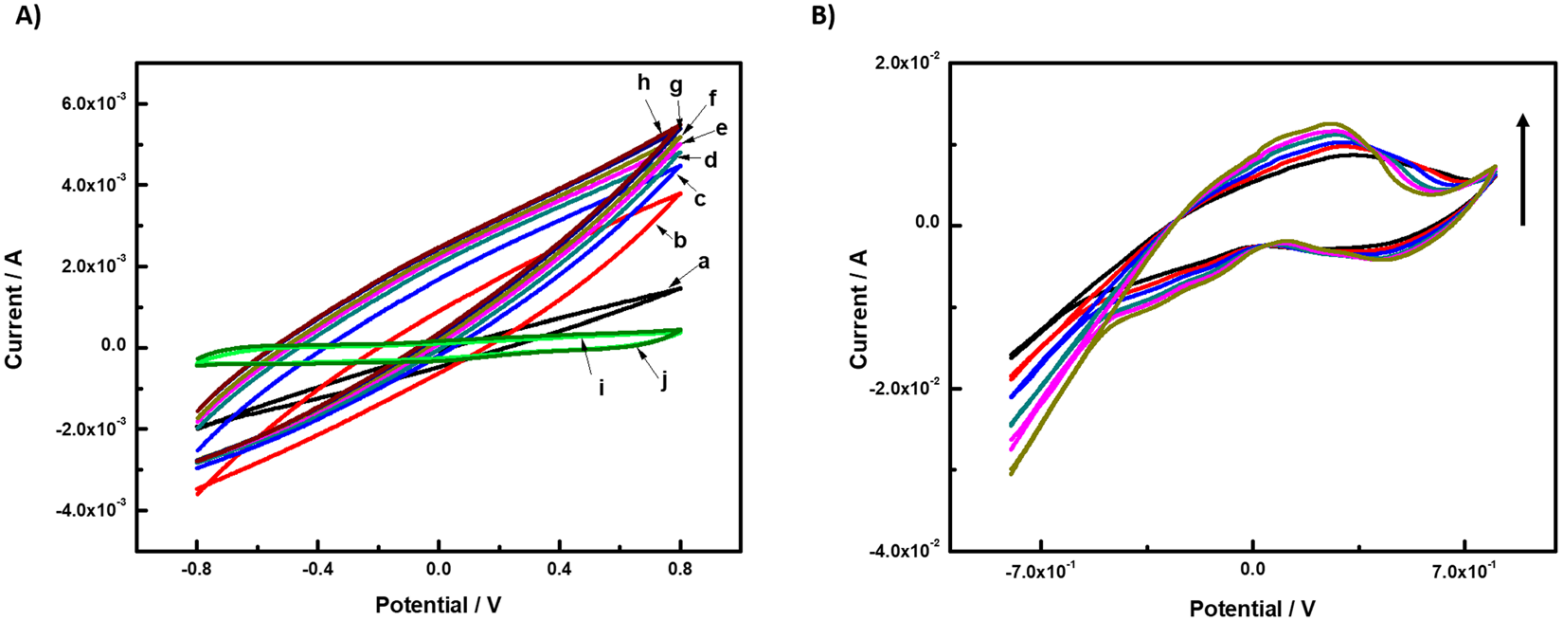
(**A**) Cyclic voltammograms (CVs) of braided Au-co-Pt wire anode in (a) 10 mM PBS solution and (b-h) 1, 3, 5, 7, 10, 15, and 20 mM glucose (pH = 7.4) and the braided Au wire in the presence of (i) 10 mM PBS solution and (j) 1 mM glucose (pH = 7.4). (**B**) Cyclic voltammograms (CVs) of braided Au-co-Pt wire anode in various concentrations of H_2_SO_4_ (0.2, 0.4, 0.6, 0.8, 1.0 and 1.2 N). The scan rate was 25 mV s^−1^. All CVs were performed at room temperature.

The colloidal Pt exhibited high coverages, wherein the electrocatyalytic activity and surface area of the Pt and Pt-free braided Au were characterized by cyclic voltammetry (CV) and scanning electron microscope (SEM), respectively. The CV of the braided Au before and after ~16 min platinization was acquired in the third cycle of the voltammograms and is shown in Fig. 2A. It is apparent that the bare braided Au wire showed little to no electrocatatytic response to 1 mM glucose. This is attributed to the fact that gold surfaces do not facilitate hydrogen adsorption^15^. Due to platinization, the CV of the Au-co-Pt in the presence of various glucose concentrations (1 mM–20 mM) exhibited a higher electrocatalytic activity than the bare braided Au. This indicates that the oxidation of glucose is associated with the interaction between gold, platinum, and the adsorbed species in the reaction, particularly the formation of adsorbed hydrogen. A comparison of the PBS solution voltammgram (a) shows that the peak charge characteristic of the platinized electrode increases with much more pronounced features and is distinguishable for anodic glucose oxidation (c-h), which is attributed to the further oxidation of gluconolactone that was produced upon the oxidation of glucose^16^ and remained adsorbed on the Au-co-Pt electrode surface. This further suggests that the gluconolactone oxidation also becomes easier on the Au-co-Pt electrodes. Although the voltammagrams of braided Au-co-Pt wire anode in PBS does not exhibit an electrochemical characteristic peak, an electrochemical characteristic peak was observed in the presence of H_2_SO_4_ (Fig. 2B) within the potential range of −0.8–0.8 V. The cyclic voltammogram of the colloidal Pt electrode in sulfuric acid solutions is characterized by the adsorption/desorption of hydrogen below 0.4 V and the oxygen-related reactions above 0.6 V^17^. However, oxygen evolution which is typically observed from 1.4 V in the anode scan could not be observed in this potential scan window. Moreover, glucose exhibit excellent reactivity on the Au-co-Pt surface, thereby facilitating the rapid current increase in the presence of 1 mM glucose where the onset of glucose oxidation was observed to be ca. −500 mV.

**Figure 3:**
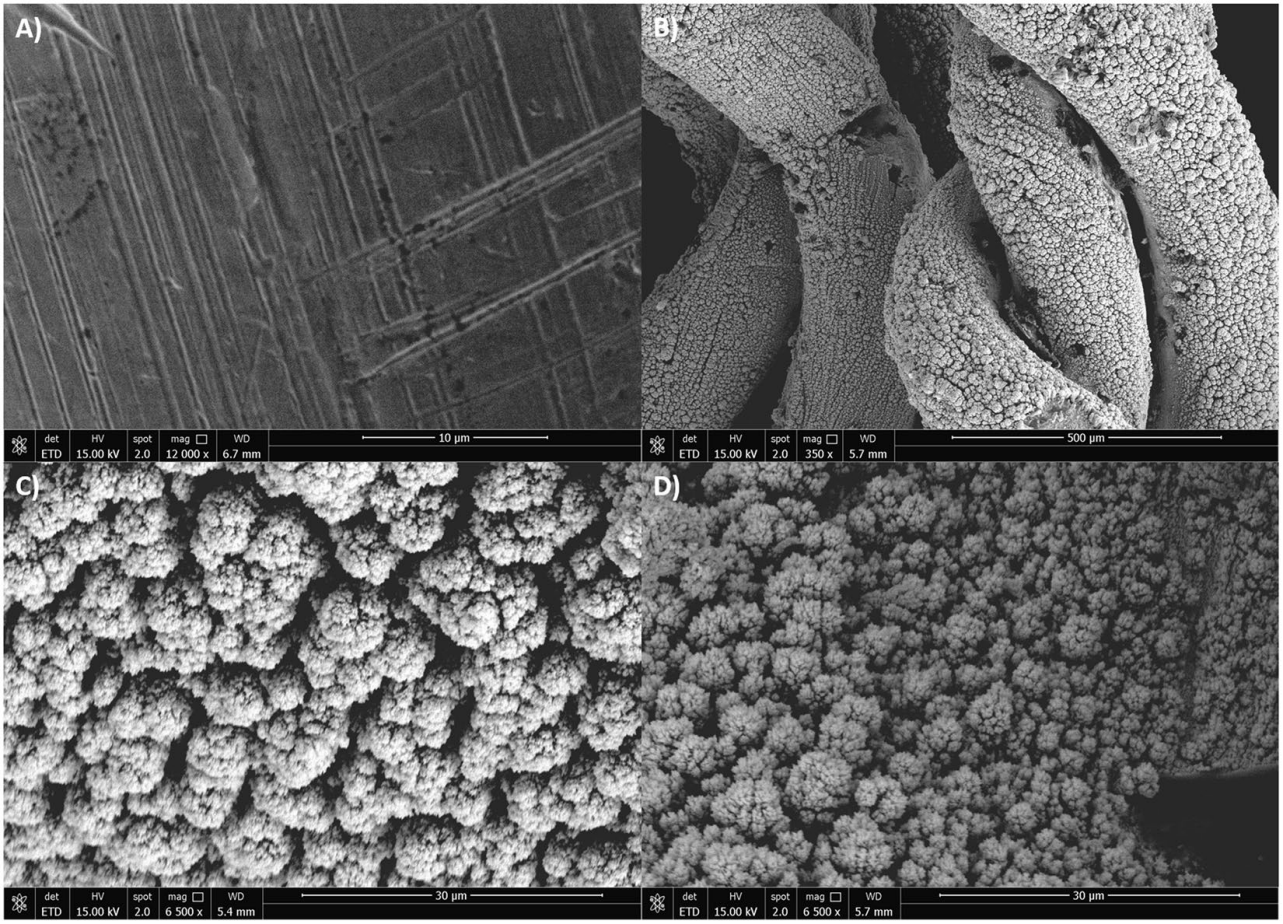
Scanning electron microscopy (SEM) images of (**A**) Au wire, (**B**) Au-co-Pt enzymeless anode and (**C**) high-resolution SEM image of Au-co-Pt enzymeless anode. SEM image of (**D**) high-resolution SEM image of Au-co-Pt after characterization in glucose solution.

As seen in the SEM micrograph of unmodified Au (Fig. 3A) and Au-co-Pt electrode (Fig. 3B–D), the surface morphology confirms the electrodeposition of colloidal platinum. Figure 3B–D suggests that most of the colloidal Pt are uniform flowerets with an average size of 7.5 ± 1.2 µm. The high resolution SEM image of the colloidal Pt (Fig. 3C,D) shows that they have nanostructures with high surface-to-volume ratios. This confirms that ~16 min deposition is enough to get at least a layer of platinum on Au. According to the SEM micrograph depicted in Fig. 3D, the Au-co-Pt anode surface that interact with the PBS and glucose solutions remained relatively intact with an overall reduction in the floweret average size (4.3 ± 0.8 µm), upon glucose oxidation at the surface of colloidal Pt on Au-wire support.

### Electrochemical characterization of BP-Au-BODx modified cathode

**Figure 4:**
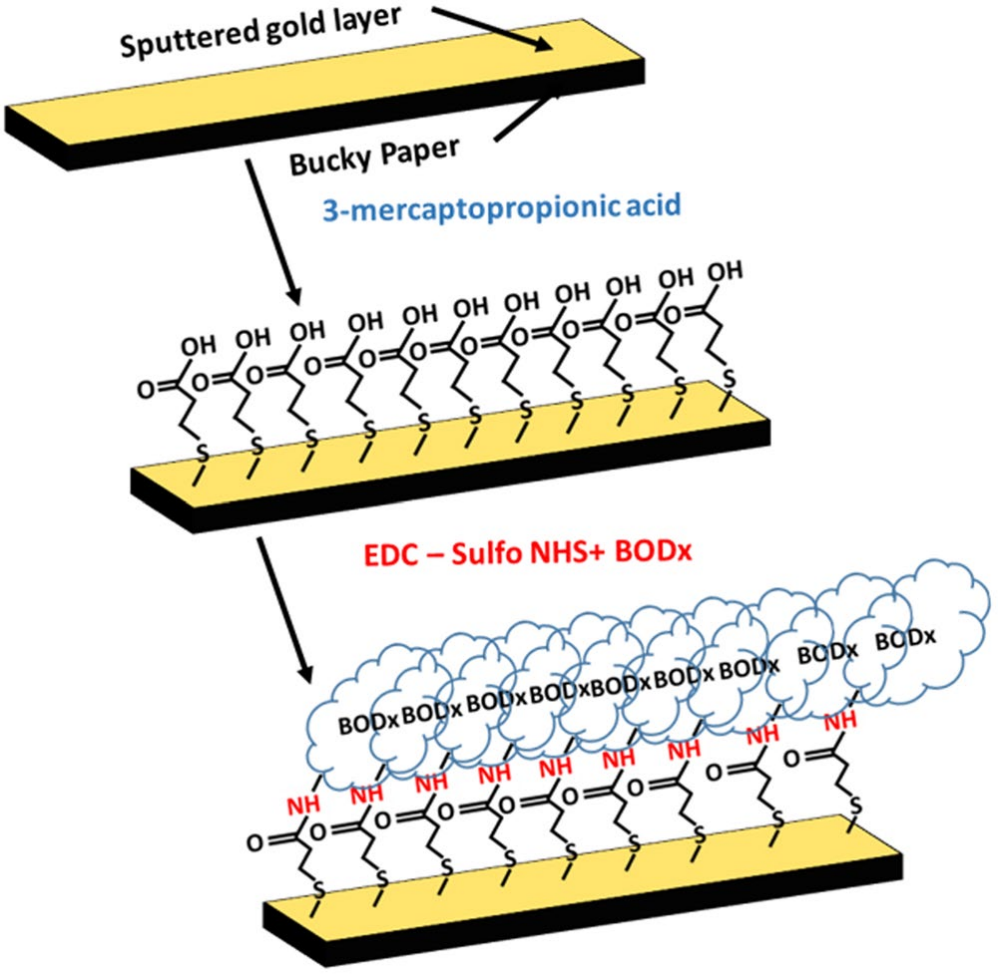
Schematic illustration of surface modification of Au coated Buckypaper electrode with bilirubin oxidase to form the BP-Au-BODx biocathode for the HBFC.

Figure 4 provides the immobilization process for BODx on Buckypaper through facile alkanethiol surface modification chemistry to create the BP-Au-BODx enzymatic cathode. The covalent immobilization of BODx on gold coated Buckypaper (BP-Au) cathodic electrode was achieved by surface modification of self-assembled monolayers (SAMs). 3-mercaptopropionic acid (MPA) was employed for the deposition of SAMs from ethanol solution onto BP-Au surface. The presence of carboxylic groups on MPA enabled the use of MPA as a molecular linker between the MWCNT network and BODx. The carboxylic groups from the BP-Au-MPA modified electrodes were first activated in EDC/Sulfo-NHS (1-Ethyl-3-(3-dimethylaminopropyl) carbodiimide hydrochloride/N-Hydroxysulfosuccinimide) solution (2:4 molar ratios) and incubated with BODx (1.25 mg/ml) to enable covalent conjugation of the amino groups located on the enzymes with the BP-MPA surface. The covalent immobilization of BODx via EDC/Sulfo-NHS on gold surfaces eliminated the non-covalent π-π stacking of pyrene on MWCNTs sidewalls. This resulted in a decreased in the loss of enzyme activity overtime at the biocathode surface, thereby leading to an effective enzyme immobilization strategy on nanotube structures without the use of pyrene derivatives. The BODx modified electrode was coated with 1% chitosan to create a microenvironment to stabilize BODx.

**Figure 5:**
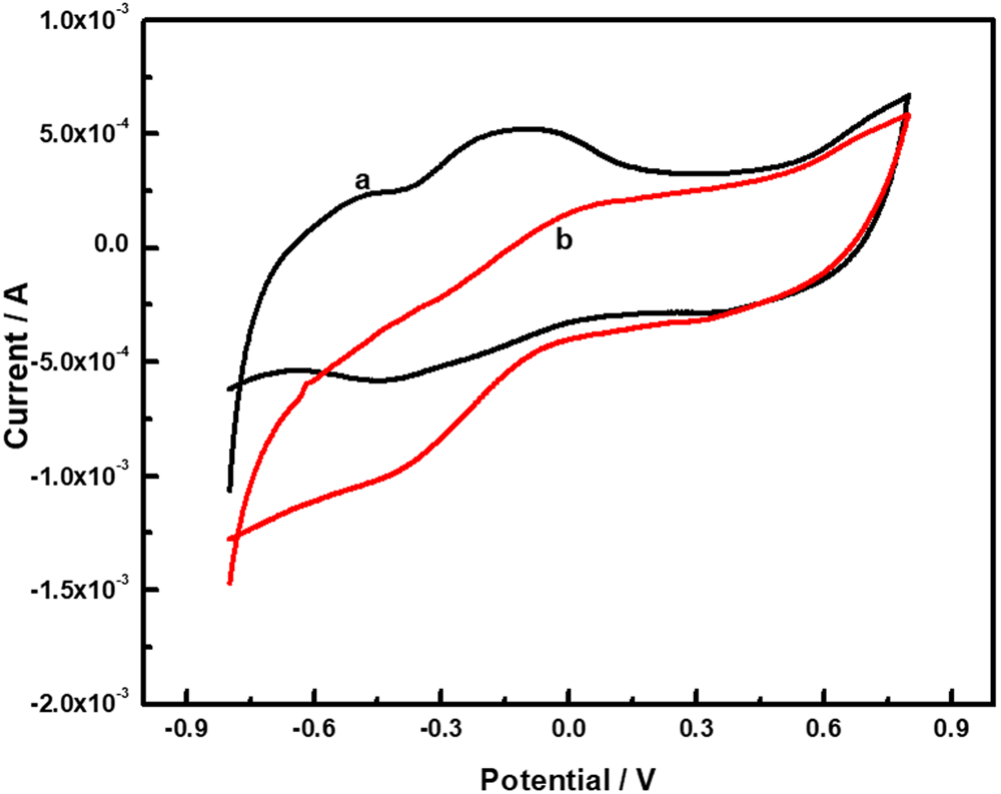
Representative cyclic voltammograms for BP-Au-BODx electrode without oxygen (**A**) and in the presence of saturated oxygen (**B**) in 10 mM PBS solution (pH = 7.4) at 25 mV s^−1^. All CVs were performed at room temperature.

In the presence of oxygen, a characteristic voltammogram for an electrocatalytic activity was depicted by the appearance of a reduction wave starting at ca. 500 mV vs. Ag/AgCl as shown in Fig. 5. The immobilized BODx on MWCNT exhibited efficient wiring of enzymes to the current collector and further confirms the successful immobilization of BODx onto the gold coated MWCNTs layer.

### Glucose/O_2_ hybrid biofuel cell characterization

Generally, glucose dehydrogenane and glucose oxidase are used to catalyze the oxidation of glucose to gluconolactone at the bioanode (Eqn. 1) and the oxygen is reduced to water by laccase or BODx enzyme at the biocathode (Eqn. 2).

1 $$Glucose\to Gluconolactone+2{H}^{+}+2{e}^{-}$$

2 $${O}_{2}+4{H}^{+}+4{e}^{-}\to 2{H}_{2}O$$

However, enzymeless anodes, in particular, those that include platinum, has been demonstrated to oxidize glucose very efficiently in neutral pH via an electrocatalytic oxidation process^18^. Nevertheless, this type of oxidation process is prone to serious poisoning due to adsorbed intermediates^19^ from the oxidation of glucose. It has been demonstrated that mitigation of the poisoning effect can be achieved by designing Pt-based bimetallic catalysts, such as Pt-Pb^20,21^, Pt-Ru^22,23^, and Pt-Au^24,25^. These electrodes showed improved electrocatalytic activity and selectivity toward glucose. Moreover, the oxidation process at the colloidal Pt surface is not fully understood and is proposed to involve a three step reaction process:

3 $$Glucose\to Glucos{e}_{ads}$$

4 $$Glucos{e}_{ads}\to Glucos{e}_{ads}+{H}_{ads}$$

5 $${H}_{ads}\to {H}^{+}+{e}^{-}$$

where the adsorbed glucose is oxidized to gluconolactone at a negative potential (−500 mV) through either a disproportionation reaction (Eqn. 4) or the oxidation of the adsorbed glucose at a more positive potential according to the cyclic voltammograms for the anode.

6 $$2Glucos{e}_{ads}\to Glucolactone+Glucos{e}_{ads}$$

This is in agreement with the expected behavior based on reports at Pt catalysts^26,27^. Au-co-Pt is observed to be a great catalyst in terms of enabling the direct electrocatalysis of glucose without the use of expensive metal substrates. A fuel cell based on platinum decorated nanoporous gold anode and a commercial Pt/C as a cathode demonstrated an open circuit voltage of 0.8 V and power density of 140 μW/cm^2^ and 180 μW/cm^2^ at 40 and 60 °C, respectively when operating in 500 mM glucose^28^. These results suggest that an Au-Pt bimetallic anode has the potential for becoming high electrical power generating fuel cell if coupled with a properly designed cathode in order to operate within the physiological glucose range (3 mM–20 mM). As demonstrated the BP-Au-BODx exhibited good electrocataylic toward oxygen reduction by facilitating direct electron transfer from the active center of BODx to the MWCNTs without the need to use redox mediators. The 3-strand braided Au-co-Pt wire anode and the BP-Au-BODx biocathode were assembled to investigate the ability of the HBFC to harvest the biochemical energy of glucose and oxygen for generating electricity.

**Figure 6:**
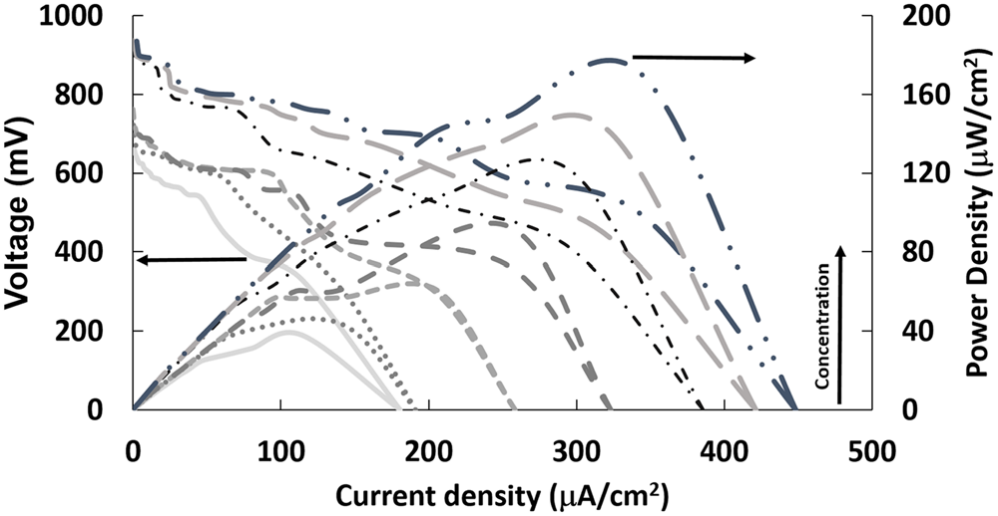
Representative polarization (solid lines) and power curves (dashed lines) obtained from 3-strand braided Au-co-Pt| BODx HBFC in the presence of various glucose concentrations (1, 3, 5, 7, 10, 15, 20 mM) generated from Au-co-Pt anode and BP-Au-BODx cathode.

The polarization and power curves for the HBFC are depicted in Fig. 6. The open circuit voltage and short circuit current density were observed to be 0.971 V and 448.18 µA/cm^2^, respectively in 20 mM glucose. The maximum power output was observed to be 169.41 µW/cm^2^ at a cell voltage of 0.491 V. At very low glucose concentration (3 mM), the open circuit voltage, short circuit current and power densities were observed to be 0.735 V, 190.99 µA/cm^2^ and 46.31 μW/cm^2^ at a cell voltage of 0.375 V, respectively.

**Figure 7:**
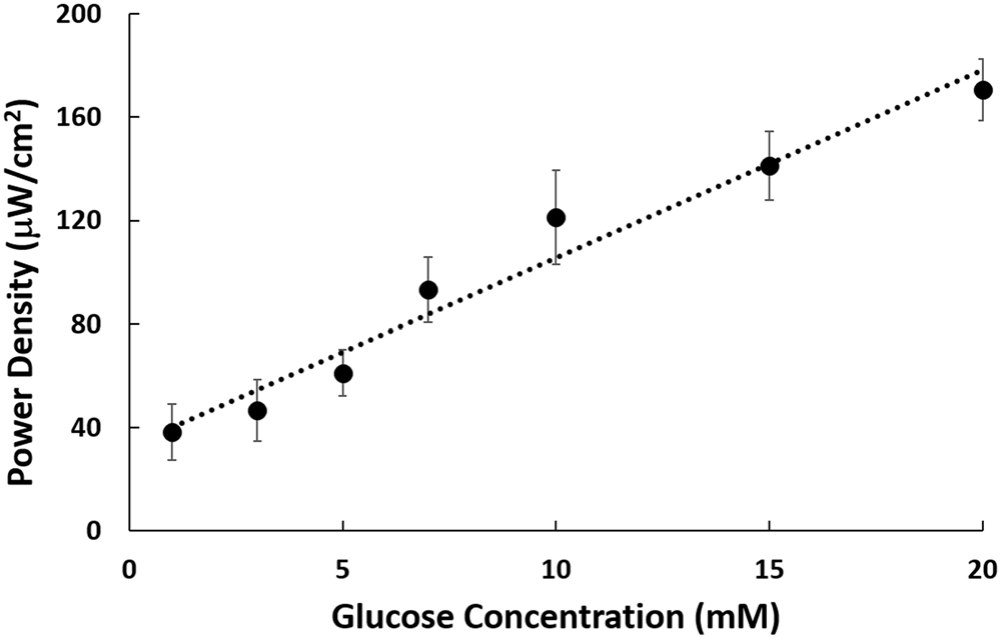
Peak power density against glucose concentration (1, 3, 5, 7, 10, 15, 20 mM) for the HBFC.

Furthermore, the peak power was observed to be directly proportional to glucose concentration as shown in Fig. 7, further depicting the behavior of the oxidation reaction of an adsorbed glucose layer upon the formation of the hydrogen adsorption layer. These results suggested that the hybrid cell exhibited a linear peak power-concentration relationship at the concentration regime ranging from 1 to 20 mM with a linear coefficient of 0.9653.

**Figure 8:**
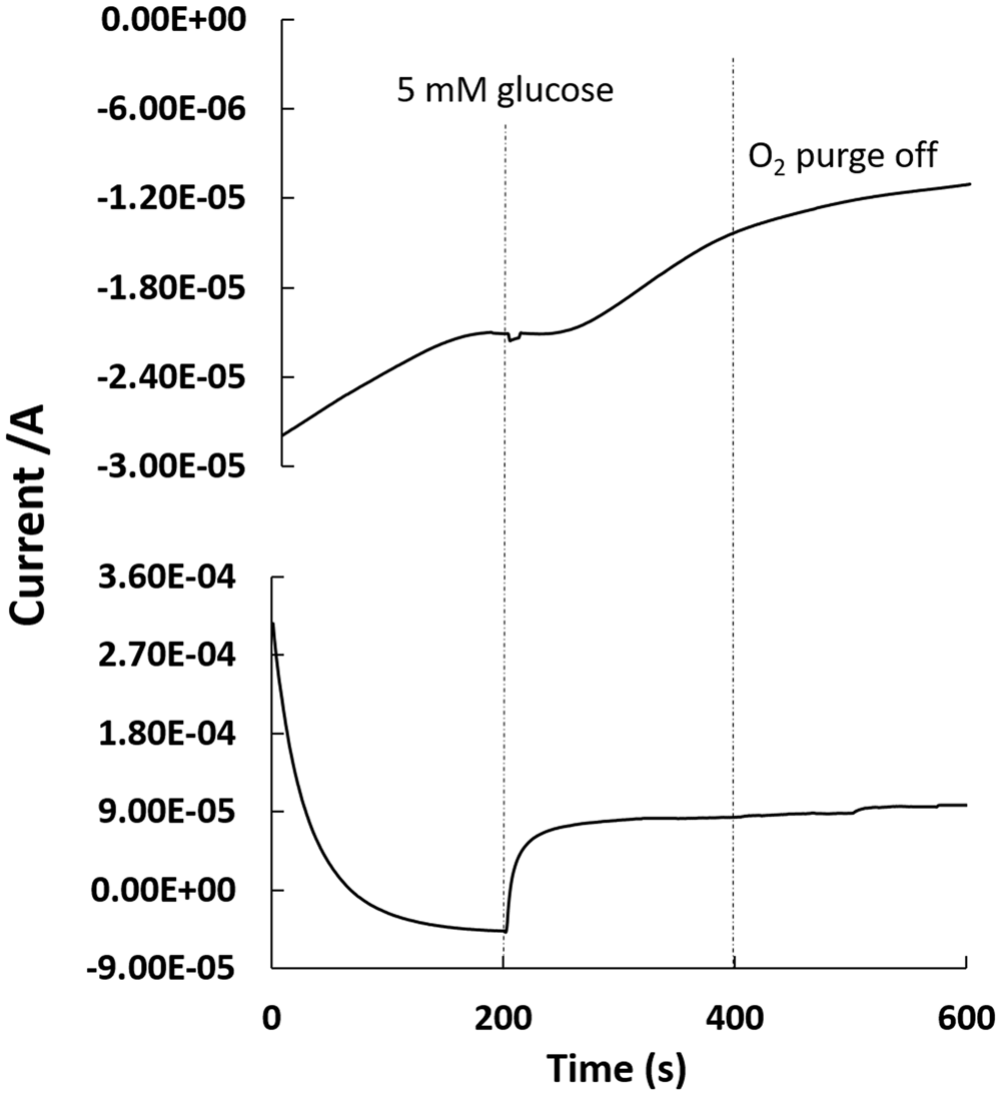
Chronoamperometric profiles of BOD biocathode (top curve) and colloidal Pt anode (bottom curve) characterized using a bipotentiostat in phosphate buffer. Oxygen was purged into the phosphate buffer at t = 0. At t = 200 s, glucose was injected to result in a final glucose concentration of 5 mM. Oxygen purging was stopped at t = 400 s.

Chronamperometric characterization was used to examined whether the byproduct of the Au-co-Pt anode or the depletion of dissolved oxygen at the biocathode resulted in fuel crossover in the hybrid biofuel cell using a bipotentiostat. A potential of 300 mV (vs. Ag/AgCl) was applied to the Au-co-Pt anode, whereas a potential of 190 mV (vs. Ag/AgCl) was applied to the BOD biocathode. Figure 8 shows the anodic current asymptotically stabilizing towards 48.8 μA in the absence of glucose. After the addition of 5 mM glucose, a sharp increase in the anodic currents was observed, thereby reflecting the electrocatalytic oxidation of glucose by the Au-co-Pt. A current of 92.7 μA was observed and this current remained stable for the entire duration of the experiment. Simultaneously, the BOD biocathode exhibited an initial current of approximately −27.7 μA. After the addition of 5 mM glucose, a small dip in the biocathdic current was observed. Since the bipotentiostatic characterization of the chronoamperometric profiles for the Au-co-Pt anode and the BOD biocathode exhibited similar characteristics in the presence of glucose and oxygen, it is concluded that the reaction at the anode does not significantly affect the reaction at the biocathode. Moreover, the hybrid biofuel cell employs Au-co-Pt and enzyme as catalysts, the HBFC is selective toward glucose and does not suffer from fuel crossover^29^. It has been shown that the use of selective catalysts allows for biofuel cells to be operated as membraneless systems that operates under mild conditions (pH 5–8, 25–37 °C), thereby making them attractive alternative to traditional fuel cells^30^.

### Power Harvesting from HBFC

**Figure 9:**
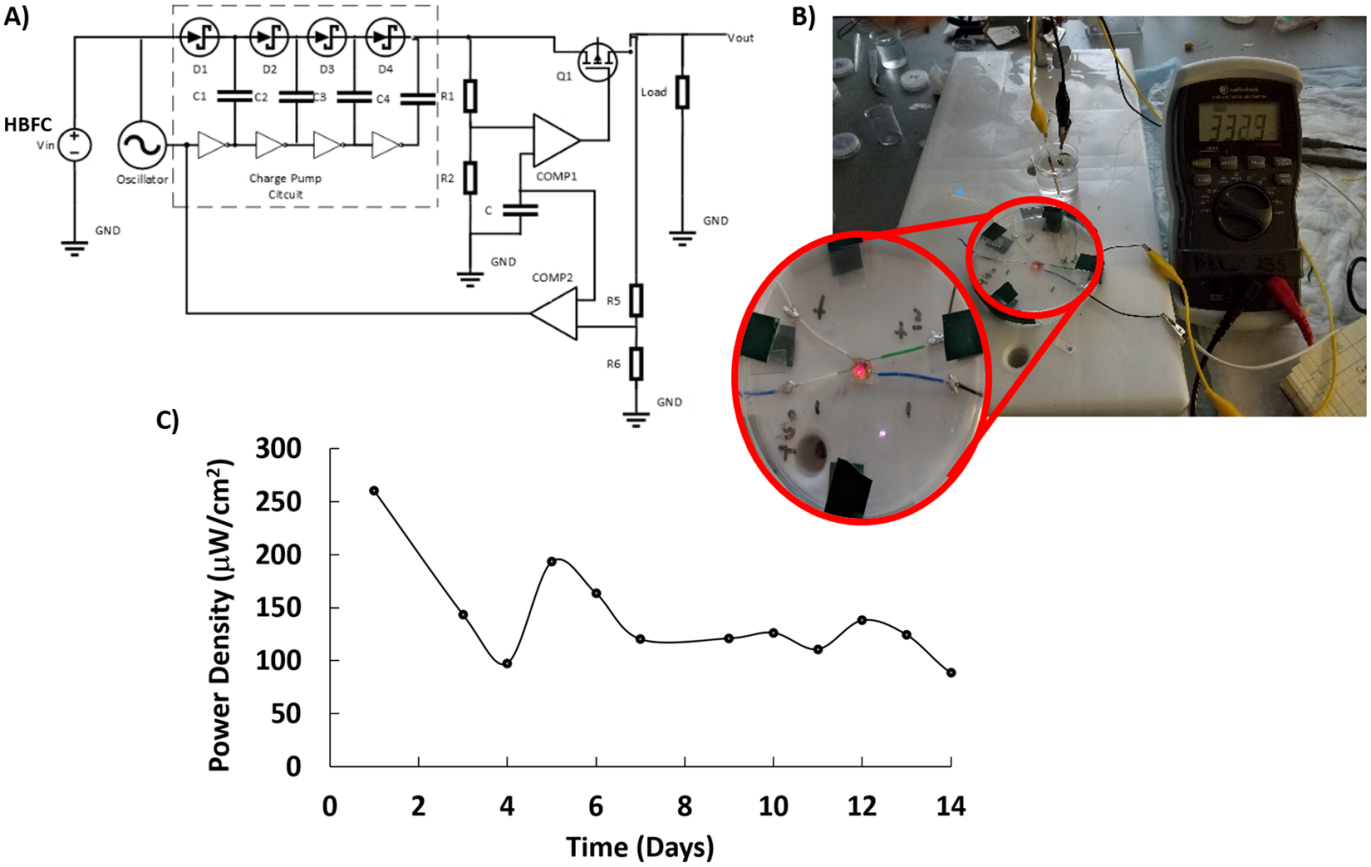
(**A**) Circuit schematic of charge pump based amplifier circuit, (**B**) Powering of a red LED using integrated HBFC-charge pump based voltage amplifier circuit, and (**C**) power curve of HBFC.

As previously mentioned, the HBFC generated a high power density of 169.41 µW/cm^2^ and an open circuit voltage as high as 0.971 V in 20 mM glucose. Hence, to explore the potential application of the Au-co-Pt|BP-Au-BODx HBFC as an energy harvester at low glucose concentration, 3 mM glucose was evaluated as the fuel source. The CV voltammogram of both anode and cathode showed onset oxidation of glucose and reduction of oxygen at a potential of −0.500 V and 0.500 V, respectively. This suggests a theoretical open circuit potential of 1 V for the membraneless HBFC. The measured open circuit voltage for 3 mM glucose was 0.735 V. The lower open circuit voltage is attributed to the losses that arise from the slow kinetics on the surface of the electrodes and the resistance to the flow of electrons and ions through the electrolytes to the surface of the electrode when the HBFC is operated. Thereby, resulting in less losses in the open circuit potentials in the presence of increasing glucose concentrations as observed in 20 mM glucose (0.971 V). Since most bioelectronic devices require an input voltage around 3 V, the standalone HBFC is incapable of driving such a system without the integration of a charge pump based voltage amplifier circuit to amplify the voltage to 1.8 V. The voltage amplification circuit consists of a charge pump, crystal oscillator, MOS transistor, active and passive elements, and energy storage elements. The voltage amplifier circuit in Fig. 9A uses a MOS transistor-switching device to control the voltage delivered to the capacitors. The capacitors are used in the start-up circuit to store and transfer energy because they are more reliable and less leakage current are observed when compared to the inductor coils used in DC-DC converters^31^. In addition, inductors can change their voltage level abruptly and to avoid the limitation of the inductive voltage converter, a capacitive solution was employed. The conversion of capacitive voltage was achieved by switching the capacitor periodically. This switching function was performed using passive diodes. A minimum input voltage of 0.275 V was required to drive the oscillation circuit of the charge pump circuit, which is readily provided by the HBFC. This input voltage is then converted to the stepped up electric power in the charge pump circuit. The stepped up electric power output from the charge pump circuit is gradually charged to the startup capacitor and the voltage of the capacitor gradually rises. Figure 9B shows the charge pump-based switching voltage amplifier circuit connected with the HBFC operating on 3 mM glucose fuel.

Long term stability and power generation is affected by several factors, wherein the integration of the biocatalyst with the appropriate high surface area physicochemical transduction element that is easy to fabricate and exhibit enhanced durability^32–36^ is highly desired to improve the efficiency of the catalyst to catalyze the reaction at the electrode surface^25^. The use of nafion^11,13,33^ and chitosan^37^ have been demonstrated to extend the functional operation of BFCs. However, generate power densities are not enough to uninterruptedly power implantable devices^11,33,38^. The first aforementioned factor remains a key challenge to overcome when employing BFCs as an implantable power source. Here the integrated energy harvesting HBFC system was used to power a red light emitting diode (LED) continuously and uninterrupted for a period of 14 days at room temperature (Fig. 8b,c) in order to assess the long term operational stability of the HBFC. In the presence of 3 mM glucose, the HBFC supplied a burst of power at the rate of 3 Hz to light the LED. The performance of the HBFC is attributed to the Pt-based bimetallic catalysts, Au-co-Pt anode acting as the dehydrogenation site wherein the Au surface facilitates the regeneration of colloidal Pt from poisoning due to adsorbed intermediates from the oxidation of glucose. The BP-Au-BODx enzyme based biocathode was observed to exhibit little or no enzyme activity after 14 days of continuous operation and this may be due to the potential denaturing of the enzyme at the biocathode surface overtime under ambient conditions. The obtained results suggest that the HBFC based on Au-co-Pt bimetallic anode and BP-Au-BODx enzyme based biocathode has the potential for becoming high power generating fuel cell if coupled with a properly designed voltage amplification circuit. This is one of the best continuously operating HBFC and most of the enzymatic biofuel cell in the literature report storage/intermittent operating stability^39–41^. This performance in continuous operational stability is presumably due to the effective oxidation of adsorbed glucose at the Au-co-Pt and reduction at the BODx modified biocathode. However, it has been observed that pieces of the colloidal Pt overtime separates from the gold surface, thereby resulting in a decrease in the electric power generated by HBFC. It is possible that the decreased in performance of the HBFC is largely a result of the loosely bound colloidal Pt. If that is the case, surface modification of 3-strand braided gold wire electrode is necessary prior to colloidal Pt deposition to roughen the gold surface and enabled intimate and strong bond between the Au and colloidal Pt, thereby increasing the overall electrocatalytic activity of the electrode for the glucose oxidation for a longer period of time. The observed data confirms that the HBFC is able to generate electricity over a long period of time to power small electronic devices.

In summary, a novel hybrid biofuel cell comprising enzymeless Au-co-Pt anode and enzyme-based BP-Au-BODx biocathode has been developed for the first time. The Au-co-Pt anode was developed by electrodeposition of colloidal Pt onto 3-strand braided Au wire for ~16 min. The deposition yielded nanostructured morphology with high surface area and high electrocatalytic activity towards glucose oxidation. The enzymatic BP-Au-BODx biocathode was developed by coating Buckypaper with a thin layer of Au to enable BODx immobilization via alkanethiol and EDC/Sulfo-NHS chemistry in order to eliminate the use of non-covalent π-π stacking of pyrene derivatives on the sidewall of MWCNT. In oxygen saturated solutions, the biocathode showed bioelectrocatalytic activity towards oxygen. This study demonstrates the importance of bioelectrode design and surface functionalization for oxidation of glucose and reduction of oxygen at the anode and biocathode, respectively. The HBFC achieved both high power density output and provided excellent performance stability in glucose concentrations as low as 3 mM. Slow release of colloidal Pt presented a severe limitation of the as prepared Au-co-Pt, thereby decreasing the performance of the HBFC system overtime. Future studies must be performed using porous/roughen Au electrodes to decrease this limitation at the anode. Considering the overall performances for hybrid biofuel cell, it can be expected that the HBFC based on Au-co-Pt and BP-Au-BODx could be applied to develop more advanced and stable energy harvesters that can operate continuously and interrupted for a long period of time to power bioelectronics devices.

## Methods

### Materials

Buckypaper, a mesh dense network of multiwalled carbon nanotubes (MWCNTs) was sputtered coated with ca. 40 nm of Au (99.9999%) to serve as the based substrate material for bilirubin oxidase (BODx) immobilization and was acquired from Nanotech labs (Yadkinville, NC). 3-mercaptopropionic acid (MPA), MES hemisodium salt, chitosan and bilirubin oxidase (BODx) derived from Myrothecium, potassium phosphate, and D-(+)−Glucose were procured from Sigma-Aldrich. 1-Ethyl-3-(3-dimethylaminopropyl) carbodiimide hydrochloride (EDC) and N-Hydroxysulfosuccinimide (sulfo-NHS) were obtained from Thermo Fisher Scientific. YSI 3140 platinizing solution was purchased from YSI Incorporated (Yellow Springs, OH). The S882z charge pump circuit from Seiko electronics.

### Electrode preparation

3-strand braided Au-wire was wrapped into a coil as shown in Fig. 1, followed by electrodeposition of colloidal Pt using three-electrode configuration in YSI 3140 platinizing solution, where Pt wire and Ag/AgCl electrodes served as the counter and reference electrodes, respectively. A potential of −225 mV was applied for ~16 min using BASi EC Epsilon potentiostat/galvanostat. After deposition, the electrode is baked at a temperature of 260 °C for 5 minutes to improve cohesion and uniformity of the platinum coating at the surface. The Buckypaper electrode material was prepared by first sputtering a thin film of gold (~ 40 nm) to yield BP-Au electrode, which was then cut into sections of 5 mm × 34 mm prior to modification with BODx. In the case of the anodic electrode, 3 strands of 7 cm gold wires (ϕ = 250 μm) were braided together to formed a Fig. 8 electrode. The BP-Au electrode was used as the substrate material for the preparation of the enzymatic cathode. The BP-Au electrode was cleaned with 2-proponal and subsequently immersed in a solution consisting of 10 mM MPA for 3 h. After the formation of self-assembled monolayers (SAMs), the BP-Au-MPA electrode was washed with 10 mM phosphate buffer solution to remove any unbound MPA. The carboxylic acid group was activated by incubating the SAMs modified electrode in EDC/sulfo-NHS (2:4, MES buffer pH 4.7) and 1.25 mg/ml BODx for 2 h. In presence of the EDC, the N-hydroxyl group of the sulfo-NHS interacts with the carboxyl groups to form reactive sites that interacts with BODx to form a strong covalent amide bond between the carboxylic group and the lysine groups on the BODx. The BODx functionalized electrode was washed with deionized water and stored in 100 mM phosphate buffer solution at 4 °C when not in use.

### Device circuit board fabrication

Computer-aided design (CAD) tool was used to design the circuit layout for the charge pump and the capacitor circuit, which is used to store the electrical power generated from the hybrid fuel cell and then release to power a small electronic device. Inkjet printing of the circuit was performed with a FujiFilm Dimatix DMP-2831 Materials Printer on bacterial nanocellulose. An aqueous ink containing a palladium-based catalyst was used. During printing, the platen temperature was set at 60 °C and the cartridge temperature was set at 70 °C. The temperatures are necessary to ensure that the ink dries out before completely penetrating the cellulose substrate. This is followed by electroless plating of a variety of metals, including copper, nickel, and gold, resulting in a metallic wiring diagram for the soldering of the circuit components.
